# Impact of admission glucose and 30-day major adverse cardiovascular events on patients with chest pain in an emergency setting: insights from the China EMPACT registry

**DOI:** 10.3389/fcvm.2024.1367704

**Published:** 2024-10-09

**Authors:** Xinxin Yin, Xin Pan, Jingyu Zhang, Shuo Wu, Weikai Cui, Yuting Wang, Chuanbao Li, Jiali Wang, Yuguo Chen

**Affiliations:** ^1^ Department of Emergency and Chest Pain Center, Qilu Hospital of Shandong University, Jinan, China; ^2^ Shandong Provincial Clinical Research Center for Emergency and Critical Care Medicine, Qilu Hospital of Shandong University, Jinan, China; ^3^ Key Laboratory of Emergency and Critical Care Medicine of Shandong Province, Qilu Hospital of Shandong University, Jinan, China; ^4^ Key Laboratory of Cardiovascular Remodeling and Function Research, Chinese Ministry of Education, Chinese National Health Commission and Chinese Academy of Medical Sciences, State and Shandong Province Joint Key Laboratory of Translational Cardiovascular Medicine, Department of Cardiology, Qilu Hospital, Cheeloo College of Medicine, Shandong University, Jinan, China

**Keywords:** acute chest pain, admission glucose, MACE, emergency department, prognosis

## Abstract

**Objective:**

Although the association between admission glucose (AG) and major adverse cardiac events (MACE) is well-documented, its relationship with 30-day MACE in patients presenting with cardiac chest pain remains unclarified. In light of this, this study aims to examine the correlation between AG levels and the incidence of MACE in patients with chest pain in an emergency setting.

**Materials and methods:**

We consecutively enrolled patients who presented to the emergency department for chest pain symptoms within 24 h from the EMPACT cohort in Eastern China (clinicaltrials.gov, Identifier: NCT02536677). The primary outcome was 30-day MACE, including all-cause death, recurrent myocardial infarction, urgent target vessel revascularization, stroke, cardiogenic shock, and cardiac arrest (CA). The associations of AG levels with 30-day MACE were analyzed using Kaplan–Meier analysis and Cox regression models.

**Results:**

Among 1,705 patients who were included in this study, 154 (9.03%) patients met the primary outcome at 30 days. The average age of the patients was 65.23 ± 12.66 years, with 1,028 (60.29%) being male and 500 (29.33%) having diabetes. The median AG levels were 7.60 mmol/L (interquartile range: 6.30–10.20). Kaplan–Meier survival analysis revealed significant differences in the 30-day MACE risk (*P* < 0.001 according to the log-rank test). We found that the highest AG level (Q4) was associated with increased MACE risk compared with the lowest AG level [adjusted hazard radio (aHR): 2.14; 95% confidence interval (CI): 1.2–3.815; *P* = 0.010]. In addition, Q4 level was also associated with increased all-cause death risk (aHR: 3.825; 95% CI: 1.613–9.07; *P* = 0.002) and increased CA risk (aHR: 3.14; 95% CI: 1.251–7.884; *P* = 0.015).

**Conclusions:**

An elevated AG level significantly correlates with a higher incidence of 30-day MACE in patients with acute chest pain. The findings reveal the importance of managing AG levels to potentially reduce the risk of adverse cardiac events.

## 1 Introduction

Chest pain is a common clinical complaint in emergency departments (EDs) across the world. Studies indicate that approximately 5%–10% of ED visits are due to chest pain, with tertiary hospitals in China reporting rates of over 20% (1–3). Various cardiovascular conditions, such as acute myocardial infarction (AMI), aortic dissection, pulmonary embolism, and others, significantly endanger patient wellbeing and survival (1, 4–6). Research has identified a 13.1% risk of major adverse cardiac events (MACE) within 30 days for patients presenting with chest pain, highlighting a considerable MACE risk in this demographic (7). Identifying individuals at high risk for MACE among those presenting with acute chest pain is therefore crucial. Diabetes mellitus is a recognized significant risk factor for cardiac chest pain (8, 9), yet the presence of diabetes alone does not sufficiently predict the likelihood of admission hyperglycemia, which can affect both diabetic and non-diabetic individuals alike.

The incidence of stress hyperglycemia is notably frequent among patients in the ED. Admission glucose (AG) is identified as the initial random blood glucose measurement taken upon hospital admission (10), serving as a stress response that can affect anyone under significant stress, especially those with critical cardiovascular conditions, trauma, or multiorgan dysfunction or failure (11). Previous research has shown its predictive value for adverse outcomes across a broad spectrum of diseases (12–14). However, the specific relationship between AG levels and prognosis in patients with acute cardiac chest pain remains unclear.

Therefore, this paper endeavors to explore the association between AG and MACE in patients experiencing acute cardiac chest pain, aiming to enhance our understanding and management of such cases.

## 2 Materials and methods

### 2.1 Study design and setting

For examining the correlation between 30-day MACE of chest pain patients and AG, we utilized data from individuals presenting with chest pain symptoms from the regional Evaluation and Management of Patients with Acute Chest Pain in China (EMPACT) cohort (*N*CT02536677). EMPACT was a multicenter prospective registry, which enrolled patients presenting to the ED with acute chest pain from 22 representative public hospitals in Shandong province, China (15). This registry contained details about clinical baseline, initial evaluation, further diagnostic testing, treatment, and other hospitalization information. Patients were tracked for MACE through medical records and telephone follow-up at 30 days after their enrollment. The final diagnoses of patients and all MACE were independently adjudicated by a clinical events committee. Finally, we excluded one hospital with a small number of AG tests from four regional grade III-A urban hospitals and ultimately selected three hospitals for inclusion in the analysis.

Patient inclusion spanned from January 2016 to October 2017 and required individuals to be over 18 years of age. These patients presented to the ED for chest pain symptoms suspected to be of cardiac origin, occurring within 24 h of pain onset. In addition, patients exhibiting atypical symptoms (such as sweating, faintness, and back pain) followed by chest pain were also considered for inclusion. Exclusions were made for patients transferred from another hospital, those previously recruited in an earlier presentation, or individuals who declined to provide informed consent.

### 2.2 Data collection and handling

As soon as a patient was identified as eligible for inclusion, research assistants collected demographic data and data on risk factors and medical history through a direct interview, as well as information from hospital records about investigations and management during hospitalization. The patient's personal information is first recorded on a standardized case report form (CRF) and then entered into the electronic data capture (EDC) system (Likangtimes Corporation, Beijing, China). The clinical data collected included those on demographics, risk factors, medical history, presenting symptoms, electrocardiograms (ECGs), medications, and in-hospital procedures.

This study adhered to the guidelines of the Declaration of Helsinki and received approval from the research ethics committee of Qilu Hospital. To ensure patient confidentiality, all identifiable personal information was anonymized before analysis. Unique identifiers were assigned to each participant to replace personal data, ensuring that no individual could be identified from the data set. Access to raw data was restricted to the primary research team, and data were stored in secure, password-protected databases. The data handling procedures were designed to comply with ethical standards and institutional guidelines. Only aggregated data were reported, ensuring that individual patient information remained confidential.

### 2.3 Measurements

After the initial screening process, a random blood sample was collected from each patient, and it was centrifuged immediately after collection. These plasma samples were analyzed at three reputed medical centers using the Johnson VITROS 5600 Integrated System, which employs dry chemical technology. We also provided unified training for the laboratory technicians of the three hospitals. The quality of sample collection and testing in each hospital is controlled by the monitoring system. In quality control, monitoring systems consist of three critical elements. First, algorithms will check for missing or implausible data; second, investigators at participating sites will verify the accuracy of all CRFs and medical records. Third, the coordinating center monitors the data online on a daily basis to confirm that they meet the project requirements.

### 2.4 Outcomes

The endpoint for this study was MACE within 30 days of patient presentation, encompassing recurrent MI, stroke, all-cause death, cardiogenic shock, urgent target vessel revascularization, and cardiac arrest (CA). The definition of recurrent MI is a new MI occurring within 30 days postadmission, defined by clinical or ECG evidence of myocardial ischemia coupled with a cardiac troponin increase and/or decrease, with at least one value exceeding the 99th percentile upper reference limit (URL), as per the fourth universal definition. Stroke is identified by acute focal neurological deficits of vascular origin, lasting ≥24 h or until death, substantiated by brain imaging (such as CT or MRI) and neurologic/neurosurgical assessments. Cardiogenic shock is defined as systolic blood pressure (SBP) <90 mmHg and/or cardiac index <2.2 L/(min m^2^) due to persistent (>30 min) cardiac dysfunction, accompanied by tissue and organ hypoperfusion. Urgent target vessel revascularization is identified by coronary revascularization for 30 days, which includes the two main types of procedures: coronary artery bypass grafting (CABG) and percutaneous coronary intervention (PCI). CA is the ejection of the heart, which suddenly stops and aortic pulsation disappears.

### 2.5 Data analysis

A descriptive analysis of the characteristics of patients with AG measurements was performed by the AG quartile group. Continuous variables were represented as means and standard deviations (SDs), and categorical variables were represented using numbers and percentages. For continuous variables, one-way ANOVA or Student's *t*-test was used, and for categorical variables, chi-square or Fisher's exact test was used. For categorical variables, the trends were calculated with the Cochran–Armitage trend test.

The dissimilarities between the quartiles in 30-day MACE were illustrated by Kaplan–Meier curves and evaluated using the log-rank test. In addition, we calculated MACE hazard ratios with 95% confidence intervals (CI) using Cox regression models. These models treated AG as either a continuous or a quartile-based categorical variable and were adjusted for potential confounders, including age, sex, body mass index (BMI), heart rate at admission, systolic blood pressure, smoking status, and past medical history (covering myocardial infarction, coronary artery disease (CAD), hypertension, diabetes mellitus, and chronic renal insufficiency), as well as diagnosis. Moreover, we also used a Chi-square test to evaluate in-hospital MACE, and this test result is included in the Supplementary Material.

Based on previous studies and results of baseline characteristics analysis, subgroup analyses were conducted across various demographics and medical histories, including age, sex, history of diabetes, hypertension, dyslipidemia, prior CAD, and the type of presenting condition (cardiac diseases and non-cardiac diseases). In this analysis, cardiac diseases included AMI, unstable angina, and stable angina, while non-cardiac diseases included arrhythmias, digestive diseases, respiratory diseases, neurodynia, and mental system diseases. Models with interaction terms were tested between AG and subgroup variables by using a likelihood ratio test, adjusting for the aforementioned covariates, unless the variable was used as a subgroup variable. A *p*-value <0.05 on a two-tailed test was considered statistically significant. Statistical analyses were conducted with SAS version 9.4 (SAS Institute, Cary, NC, USA).

## 3 Results

### 3.1 Characteristics of the study subjects

In this study, 1,705 patients from three hospitals (Qilu Hospital, Jinan, China; Zibo Central Hospital, Zibo, China; the Affiliated Hospital of Jining Medical College, Jining, China) met the inclusion criteria. The cohort comprised 1,705 individuals presenting with chest pain, with an average age of 65.23 ± 12.66 years. Of these patients, 1,028 (60.29%) were male, and 500 (29.33%) had a history of diabetes. The median AG levels were 7.60 mmol/L, with an interquartile range of 6.30–10.20 mmol/L. We divided the AG levels into quartiles: Q1, 5.7 (5.3, 6) mmol/L; Q2, 6.8 (6.6, 7.1) mmol/L; Q3, 8.5 (7.9, 9.2) mmol/L; Q4, 13.2 (11.5, 15.8) mmol/L.

All included patients with chest pain comprised 1,213 patients with cardiac diseases (802 cases of AMI, 379 cases of unstable angina, and 32 cases of stable angina) and 492 patients with non-cardiac diseases (56 cases of arrhythmias, 33 cases of digestive disease, 19 cases of disease of respiratory diseases, 46 cases of artery diseases, 19 cases of neuromuscular diseases, 12 cases of mental system diseases, 204 cases of chest pain with unknown reason, and 103 cases of others).

Notably, we observed distinct differences in baseline characteristics among the quartiles, including variations in age (*P* < 0.001), sex (*P* = 0.009), heart rate at admission (*P* < 0.001), history of hypertension (*P* < 0.001), history of diabetes (*P* < 0.001), history of dyslipidemia (*P* = 0.047) and disease classification (*P* < 0.001) (cardiac vs. non-cardiac diseases). Moreover, the use of ADP receptor antagonists also varied between these groups (*P* < 0.001) (Table 1). In addition, an increasing trend was evident in the parameters of age (*P* < 0.001), heart rate at admission (*P* < 0.001), prevalence of prior diabetes (*P* < 0.001), and disease classification (*P* = 0.003) as AG levels rose.

**Table 1:** Baseline characteristics by admission glucose quartiles.

Characteristic	Q1 (*N* = 414)	Q2 (*N* = 425)	Q3 (*N* = 442)	Q4 (*N* = 424)	*P*	*P* for trend
Demographics						
Age, years	63.19 ± 13.90	65.27 ± 12.48	65.84 ± 12.24	66.55 ± 11.74	<0.001	<0.001
Men, *n* (%)	272 (65.70)	266 (62.59)	256 (57.92)	234 (55.19)	0.009	0.275
Risk factor						
BMI, kg/m^2^	24.89 ± 3.60	25.14 ± 3.32	25.31 ± 3.98	25.37 ± 3.48	0.223	0.223
HR, bpm	75.37 ± 17.12	77.96 ± 19.76	81.06 ± 22.97	85.61 ± 21.92	<0.001	<0.001
SBP, mmHg	148.20 ± 26.50	143.94 ± 28.29	144.37 ± 29.23	146.85 ± 31.31	0.100	0.100
Current smoking, *n* (%)	105 (25.36)	103 (24.24)	119 (26.92)	96 (22.64)	0.518	0.409
Medical history, *n* (%)						
Prior MI	91 (21.98)	86 (20.24)	90 (20.36)	105 (24.76)	0.342	0.931
Prior CAD	139 (33.57)	124 (29.18)	124 (28.05)	146 (34.43)	0.112	0.515
Chronic renal dysfunction	11 (2.66)	8 (1.88)	9 (2.04)	15 (3.54)	0.401	0.814
Hypertension	234 (56.52)	247 (58.12)	251 (56.79)	294 (69.34)	<0.001	0.104
Diabetes	28 (6.76)	53 (12.47)	116 (26.24)	303 (71.46)	<0.001	<0.001
Dyslipidemia	31 (7.49)	33 (7.76)	42 (9.50)	53 (12.5)	0.047	0.559
Disease classification, *n* (%)						
Cardiac diseases	254 (61.35)	293 (68.94)	325 (73.53)	341 (80.42)	<0.001	0.003
Non-cardiac diseases	160 (38.65)	132 (31.06)	117 (26.47)	83 (19.58)		
Medical test in the emergency department, *n* (%)						
ECG	400 (96.62)	403 (94.82)	419 (94.80)	411 (96.93)	0.247	0.469
cTnI	260 (62.80)	244 (57.41)	275 (62.22)	259 (61.08)	0.372	0.102
Medication in the emergency department, *n* (%)						
Aspirin	258 (62.32)	282 (66.35)	297 (67.19)	294 (69.34)	0.183	0.173
ADP receptor antagonists	194 (46.86)	224 (52.71)	261 (59.05)	254 (59.91)	<0.001	0.101
Statin	175 (42.27)	165 (38.82)	182 (41.18)	173 (40.80)	0.781	0.317
Thrombolysis	2 (0.52)	1 (0.25)	3 (0.70)	2 (0.48)	0.878	0.489

BMI, body mass index; HR, heart rate; SBP, systolic blood pressure; MI, myocardial infarction; CAD, coronary artery disease; ECG, electrocardiogram; ADP, adenosine diphosphate.

For continuous variables, one-way ANOVA or Student's *t*-test was used, and for categorical variables, a chi-square or Fisher's exact test was used. For categorical variables, the trends were calculated using the Cochran–Armitage trend test. AG level (mmol/L): Q1, 5.7 (5.3, 6); Q2, 6.8 (6.6, 7.1); Q3, 8.5 (7.9, 9.2); Q4, 13.2 (11.5, 15.8).

### 3.2 Association between AG levels and the risk of 30-day MACE in patients with chest pain

**Figure 1:**
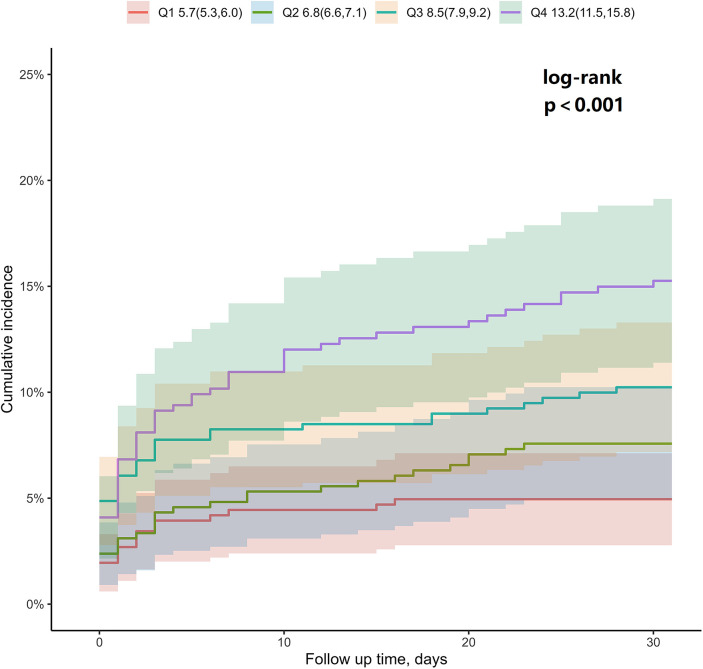
A Kaplan–Meier survival analysis of 30-day MACE according to admission glucose levels in patients with chest pain. The shadow area represents the survival interval of the Kaplan–Meier curve. MACE, major adverse cardiac events.

During the follow-up, MACE occurred in 154 patients, accounting for 9.03% of all patients (Table 2). Notably, the Kaplan–Meier curves depicted in Figure 1 demonstrate that there is a significant difference in the risk of MACE among the quartile groups (*P* < 0.001 according to the log-rank test). The shadow area in this figure represents the survival interval of the Kaplan–Meier curve. Furthermore, there were significant differences in the risk of all-cause mortality (*P* < 0.001), CA (*P* = 0.008), cardiogenic shock (*P* = 0.014), and stroke (*P* = 0.032) among patients in the quartile groupings, while no differences were found in the risk of MI (*P* = 0.370) and urgent target vessel revascularization (*P* = 0.580) (Supplementary Figure S1).

**Table 2:** Adjusted hazard ratios (95% CI) of 30-day MACE according to quartiles of admission glycemia levels in patients with chest pain.

Events	*n*/*N*	Q1 (*N* = 414)	Q2 (*N* = 425)		Q3 (*N* = 442)		Q4 (*N* = 424)		Continuous glucose	
			aHR (95% CI)	*P*	aHR (95% CI)	*P*	aHR (95% CI)	*P*	aHR (95% CI)	*P*
30-day MACE	154/1,705	Ref	1.282 (0.724–2.272)	0.395	1.424 (0.822–2.466)	0.207	2.14 (1.2–3.815)	0.010	1.083 (1.046–1.122)	<0.001
All-cause death	67/1,705	Ref	0.942 (0.338–2.624)	0.909	1.909 (0.815–4.474)	0.137	3.825 (1.613–9.07)	0.002	1.144 (1.094–1.196)	<0.001
MI	19/1,705	Ref	3.960 (0.811–19.333)	0.089	1.863 (0.323–10.732)	0.486	2.391 (0.398–14.359)	0.340	0.997 (0.878–1.131)	0.957
Cardiogenic shock	83/1,705	Ref	1.107 (0.506–2.422)	0.799	1.346 (0.65–2.789)	0.423	1.493 (0.673–3.31)	0.324	1.05 (0.997–1.106)	0.062
CA	64/1,705	Ref	1.763 (0.698–4.453)	0.230	1.679 (0.678–4.16)	0.263	3.14 (1.251–7.884)	0.015	1.12 (1.061–1.182)	<0.001

MACE, major adverse cardiovascular events; aHR, adjusted hazard ratios; Major Adverse Cardiovascular Events; MI, myocardial infarction; CA, cardiac arrest.

Urgent target vessel revascularization and stroke were not included in the analysis because the number of events was less than 10. The adjustment for confounding factors includes age, gender, BMI, heart rate at admission, systolic blood pressure, smoking status, and past medical history (including myocardial infarction, coronary artery disease, hypertension, diabetes, and chronic renal insufficiency), as well as diagnosis. AG level (mmol/L): Q1, 5.7 (5.3, 6); Q2, 6.8 (6.6, 7.1); Q3, 8.5 (7.9, 9.2); Q4, 13.2 (11.5, 15.8).

After performing Cox regression analysis, we found that the Q4 AG levels were significantly associated with an increased risk of MACE [adjusted hazard radio (aHR): 2.14; 95% CI: 1.2–3.815; *P* = 0.010]. Furthermore, the Q4 AG levels also correlated with an elevated risk of all-cause death (aHR: 3.825; 95% CI: 1.613–9.07; *P* = 0.002) and CA (aHR: 3.14; 95% CI: 1.251–7.884; *P* = 0.015). However, no statistically significant association was observed for the 30-day risk of recurrent MI and cardiogenic shock between the groups (aHR: 2.391; 95% CI: 0.398–14.359; *P* = 0.340; aHR: 1.493; 95% CI: 0.673–3.31; *P* = 0.324, respectively). In addition, AG as a continuous variable was associated with increased risks of 30-day MACE (aHR: 1.083; 95% CI: 1.046–1.122; *P* < 0.001), all-cause death (aHR: 1.144; 95% CI: 1.094–1.196; *P* < 0.001), and CA (aHR: 1.12; 95% CI: 1.061–1.182; *P* < 0.001).

We also analyzed AG levels and MACE during ED admission and hospitalization. MACE during ED admission and hospitalization also represents MACE in the emergency phase. In our analysis, a total of 129 patients experienced MACE in the ED and during hospitalization. The median length of stay for patients in both the ED and the hospital was 10 days (range: 7–14 days). Finally, we found that patients with high AG levels had a higher likelihood of experiencing MACE, all-cause death, MI, cardiogenic shock, CA, and stroke during their ED visits and hospital stays (Supplementary Table S1).

### 3.3 Subgroup analysis of included patients

Subgroup analysis was conducted based on age (over 65 years old), sex, previous medical history (diabetes, hypertension, dyslipidemia, and CAD), and the type of diagnosis. The results indicated that AG levels were significantly correlated with 30-day MACE, regardless of the patient's age, history of diabetes, hypertension, or CAD (Table 3). In addition, AG was significantly associated with 30-day MACE in male (aHR: 1.09; 95% CI: 1.045–1.137; *P* < 0.001) normolipidemic patients (aHR: 1.082; 95% CI: 1.043–1.122; *P* < 0.001) and cardiac disease patients (aHR: 1.081; 95% CI: 1.039–1.124; *P* < 0.001). Intriguingly, no notable interaction was found between each subgroup and AG levels in the overall cohort analysis.

**Table 3:** A subgroup analysis between admission glucose levels and 30-day MACE in patients with chest pain.

Subgroup	*n*/*N*	aHR (95% CI)	*P*	*P* for interaction
Age				0.635
≤65	60/862	1.102 (1.041–1.166)	0.001	
>65	94/843	1.080 (1.030–1.132)	0.001	
Sex				0.780
Male	103/1,028	1.09 (1.045–1.137)	<0.001	
Female	51/677	1.064 (0.997–1.136)	0.062	
Diabetes				0.668
No	98/1,205	1.092 (1.018–1.17)	0.013	
Yes	56/500	1.081 (1.035–1.128)	*P* < 0.001	
Hypertension				0.994
No	62/679	1.074 (1.009–1.144)	0.026	
Yes	92/1,026	1.091 (1.042–1.142)	*P* < 0.001	
Dyslipidemia				0.740
No	132/1,546	1.082 (1.043–1.122)	*P* < 0.001	
Yes	22/159	0.955 (0.797–1.145)	0.618	
Prior CAD				0.871
No	113/1,172	1.070 (1.025–1.117)	0.002	
Yes	41/533	1.097 (1.032–1.166)	0.003	
Diagnosis				0.474
Cardiac diseases	127/1,213	1.081 (1.039–1.124)	<0.001	
Non-cardiac diseases	27/492	1.055 (0.972–1.146)	0.203	

MACE, major adverse cardiovascular events; aHR, adjusted hazard ratios; CAD, coronary artery disease.

AG level (mmol/L): Q1, 5.7 (5.3, 6); Q2, 6.8 (6.6, 7.1); Q3, 8.5 (7.9, 9.2); Q4, 13.2 (11.5, 15.8).

## 4 Discussion

In this registry-based cohort study, 1,705 patients discharged after treatment for acute cardiac chest pain from three tertiary hospitals in Eastern China were enrolled. The rate of incidence of 30-day MACE was 9.03%. The study's findings suggest that patients with elevated AG levels have a higher risk of experiencing 30-day MACE compared with those with lower levels of AG. Moreover, Cox regression analysis, adjusted for confounding factors, confirmed that AG levels were significantly associated with 30-day MACE. Particularly, the risk of MACE was notably higher among male patients who were diagnosed with cardiac diseases. The findings also indicate that regardless of whether a patient is over 65 years old, and has comorbidities such as hypertension, CAD, and diabetes, AG levels are significantly associated with 30-day MACE. However, no significant interaction between AG levels and each subgroup with regard to the risk of MACE was observed.

Despite previous research exploring the correlation between AG and various specific diseases, such as AMI (16, 17) and other critical diseases (18–21), the role of AG in patients with undifferentiated chest pain remains unknown. Considering the importance of managing patients with chest pain in emergency settings, clarifying the predictive role of AG would have clinical significance. Previous studies have found that AG levels are significantly correlated with both long-term and short-term prognoses of acute critical illnesses (17, 21), while this observational study examined the relationship between AG and 30-day MACE of patients with acute chest pain. As an indication of stress-induced hyperglycemia, our findings demonstrate that AG serves as a strong prognostic indicator for short-term outcomes in individuals experiencing chest pain. Numerous factors contribute to the development of stress hyperglycemia, such as inflammation, heightened release of counterregulatory hormones, and the suppression of pancreatic beta cells, as well as interventions like the administration of glucocorticoids or parenteral nutrition (22, 23). Moderate stress hyperglycemia has been found to enhance cell survival factors and decrease apoptosis, leading to a potential reduction in infarct size and improvement in systolic function in cardiogenic diseases (24). However, excessive stress hyperglycemia is associated with oxidative stress, inflammatory responses, cell damage to coronary microcirculation, and significantly impaired cardioprotective responses. In addition, the aggregation of platelets in response to ADP triggers the production of plasma catecholamine, which further exacerbates microcirculation dysfunction and thrombogenesis (25–27).

In examining the relationship between AG levels and 30-day MACE, we further carried out subgroup analysis. We found that AG levels were significantly correlated with 30-day MACE, regardless of the patient's age, history of diabetes, hypertension, or CAD. In the case of other diseases, many researchers have studied the relationship between AG levels and outcomes in males or females, but the results are varied (28–30). Takada et al. (30) found that the rate of mortality was higher in hyperglycemic men compared with lower blood glucose male and female groups, but there were no differences between women groups in respect to glycemia after adjustment for coronary risk factors, which is consistent with our findings. We found that AG levels were associated with 30-day MACE in men but not in women. In addition, we found a significant association between AG levels and outcomes in patients over 65 years, which is consistent with the findings of previous studies of AMI (31). Mamadjanov et al. (31) further grouped patients over the age of 65 and found that AG was significantly associated with 28-day case fatality in patients with AMI who were aged 65–74 years but not 75–84 years. Furthermore, in our subgroup analysis, non-diabetic patients with elevated AG levels exhibited a higher aHR compared with diabetic patients (aHR: 1.87; 95% CI: 0.932–3.752; *P* = 0.078 for non-diabetics; aHR: 1.443; 95% CI: 0.335–6.22; *P* = 0.623 for diabetics), despite *p*-values exceeding 0.05. This could be attributed to a significant proportion of non-diabetic patients having underlying insulin resistance, thereby increasing their mortality risk (32, 33).

In this analysis, there were only 492 patients with non-cardiac diseases, among whom 27 patients experienced 30-day MACE. Ultimately, we found no correlation between AG levels and 30-day MACE in patients with non-cardiac diseases. However, previous studies have shown that patients with pulmonary embolism in the fourth AG quartile, regardless of their diabetes history, had a significantly higher all-cause and PE-cause in-hospital mortality compared with those in the first quartile (34). In addition, among patients with diabetes and pulmonary embolism, those with higher AG levels tend to have a higher proportion of massive and submassive pulmonary embolism and higher pro-BNP levels (35). Furthermore, in other non-cardiac diseases such as aortic dissection, AG levels are also associated with poor patient outcomes (36). Therefore, we feel that larger sample size studies are needed in the future to validate the correlation between AG levels and 30-day MACE in patients with non-cardiac diseases.

Moreover, extensive previous research has highlighted the advantages of strict blood glucose control (37, 38). Our study underscores the importance of considering AG as a critical predictor in the ED. Physicians should particularly focus on chest pain patients with elevated AG levels, initiating treatment for early control of stress hyperglycemia. In addition to the above, previous studies did not evaluate how the relationship between AG levels and outcomes varies between different medical history subgroups in other diseases.

This study has several limitations. First, it did not investigate the issue of glycemic management of patients postadmission, nor could it elaborate on the correlation between in-hospital glucose levels and prognosis. Future research could further explore this aspect and the relationship between various tests and patients with chest pain. Second, the study included patients only from three hospitals in Eastern China, and therefore, it may not be applicable to other regions. Third, although we included diabetes history as a confounder for adjusted hazard ratios and performed a subgroup analysis of diabetes history, we did not collect the test values of glycated hemoglobin A1c (HbA1c). Last, being an observational study, the potential for the presence of unmeasured confounders remains, despite our efforts to adjust for known confounders.

## 5 Conclusion

The present study investigates the relationship between AG levels and the incidence of MACE within a 30-day period in patients presenting with cardiac chest pain in an emergency setting. The results demonstrate that patients with elevated AG levels face an increased risk of MACE within the aforementioned time frame. Therefore, it is imperative for emergency physicians to prioritize the monitoring and management of glucose levels in patients with chest pain, especially those presenting with high AG levels, as early as possible.

## Data Availability

The original contributions presented in the study are included in the article/Supplementary Materials, and further inquiries can be directed to the corresponding authors.
