# Supplementary material for: Impact of admission glucose and 30-day major adverse cardiovascular events on patients with chest pain in an emergency setting: insights from the China EMPACT registry
Source: Front Cardiovasc Med. 2024 Oct 9;11:1367704. doi: 10.3389/fcvm.2024.1367704 (PMC11496057; doi:10.3389/fcvm.2024.1367704)
Supplement: Supplementary file 1 [file Datasheet1.docx]

Supplementary Material

# Supplementary Figures and Tables

For more information on Supplementary Material and for details on the different file types accepted, please see [here](https://www.frontiersin.org/guidelines/author-guidelines#supplementary-material).

## Supplementary Figure


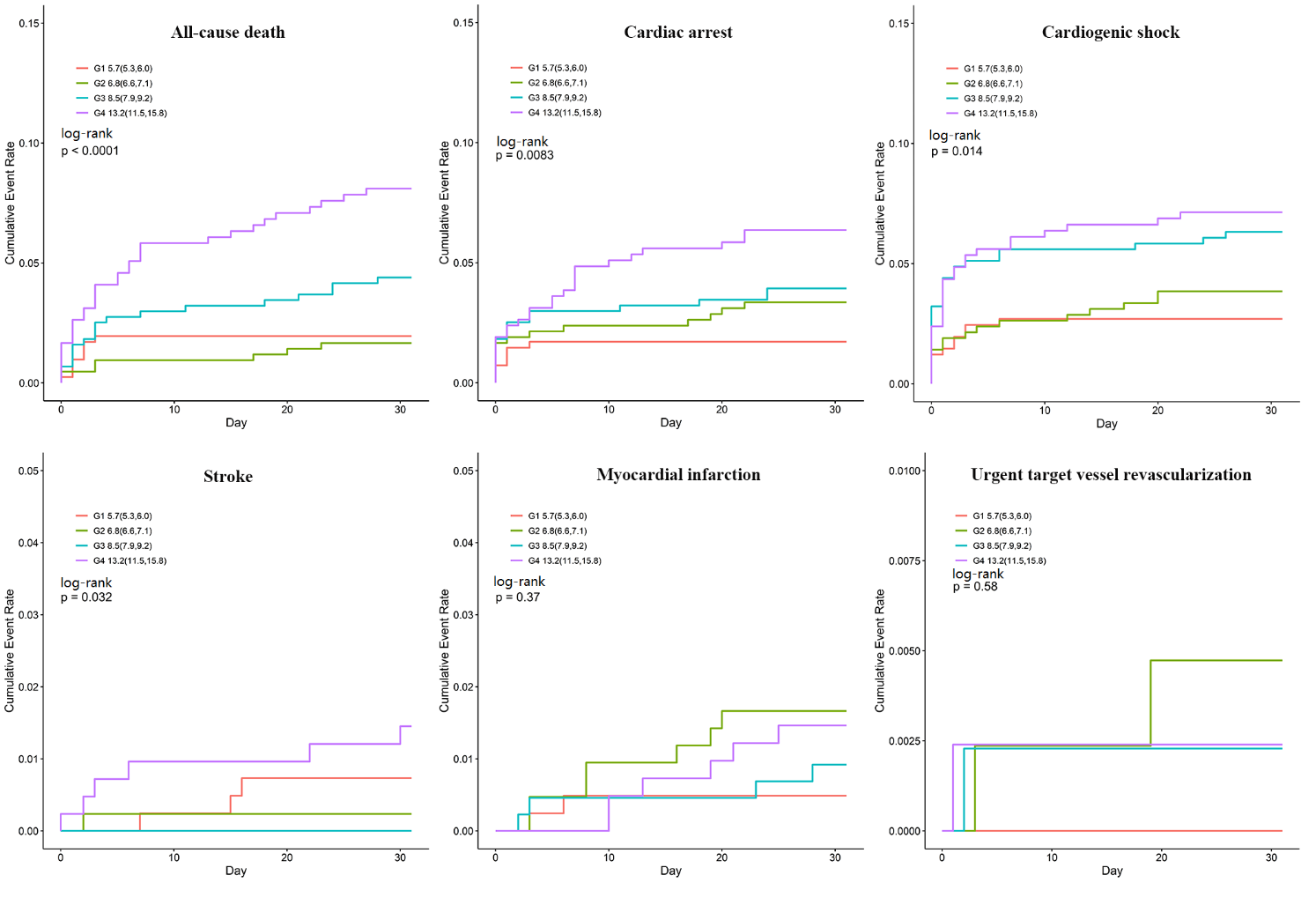


**Supplementary Figure 1. Kaplan–Meier survival analysis of 30-day events according to admission glucose levels in chest pain patients**

## Supplementary Table

**Supplementary Table 1 Comparison between 30-day outcomes and** **in-hospital outcomes according to admission glucose levels**

| **Variables** | **Q1 (N=414)** | **Q2 (N=425)** | **Q3 (N=442)** | **Q4(N=424)** | **All** | **P** |
| --- | --- | --- | --- | --- | --- | --- |
| **30-day outcomes** |  |  |  |  |  |  |
| MACE | 20(4.83) | 31(7.29) | 43(9.73) | 60(14.15) | 154(9.03) | <0.001 |
| All-cause death | 8(1.93) | 7(1.65) | 19(4.3) | 33(7.78) | 67(3.93) | <0.001 |
| MI | 2(0.48) | 7(1.65) | 4(0.9) | 6(1.42) | 19(1.11) | 0.349 |
| Urgent target vessel revascularization | 0(0) | 2(0.47) | 1(0.23) | 1(0.24) | 4(0.23) | 0.757 |
| cardiogenic shock | 11(2.66) | 16(3.76) | 27(6.11) | 29(6.84) | 83(4.87) | 0.015 |
| CA | 7(1.69) | 14(3.29) | 17(3.85) | 26(6.13) | 64(3.75) | 0.008 |
| Stroke | 3(0.72) | 1(0.24) | 0(0) | 6(1.42) | 10(0.59) | 0.019 |
| **Outcomes during ED and hospital stays** | | | | | | |
| MACE | 18(4.35) | 22(5.18) | 35(7.92) | 54(12.74) | 129(7.57) | <0.001 |
| All-cause death | 7(1.69) | 5(1.18) | 15(3.39) | 26(6.13) | 53(3.11) | <0.001 |
| MI | 1(0.24) | 3(0.71) | 0(0) | 6(1.42) | 10(0.59) | 0.022 |
| Urgent target vessel revascularization | 0(0) | 1(0.24) | 0(0) | 1(0.24) | 2(0.12) | 0.618 |
| cardiogenic shock | 10(2.42) | 13(3.06) | 25(5.66) | 29(6.84) | 77(4.52) | 0.005 |
| CA | 6(1.45) | 12(2.82) | 17(3.85) | 26(6.13) | 61(3.58) | 0.003 |
| Stroke | 2(0.48) | 0(0) | 0(0) | 5(1.18) | 7(0.41) | 0.009 |

MACE, Major Adverse Cardiovascular Events; MI, Myocardial Infarction; CA, Cardiac Arrest. ED, emergency department. AG level (mmol/L): Q1, 5.7(5.3,6); Q2, 6.8(6.6,7.1); Q3, 8.5(7.9,9.2); Q4, 13.2(11.5,15.8).
